# Granzyme B PET Imaging Stratifies Immune Checkpoint Inhibitor Response in Hepatocellular Carcinoma

**DOI:** 10.1155/2021/9305277

**Published:** 2021-12-09

**Authors:** Julian L. Goggi, Boominathan Ramasamy, Yun Xuan Tan, Siddesh V. Hartimath, Jun Rong Tang, Peter Cheng, Rasha Msallam, Ann-Marie Chacko, You Yi Hwang, Edward G. Robins

**Affiliations:** ^1^Institute of Bioengineering and Bioimaging, Agency for Science, Technology and Research (A^∗^STAR), 11 Biopolis Way, #01-02 Helios, Singapore 138667; ^2^Laboratory for Translational and Molecular Imaging (LTMI), Cancer and Stem Cell Biology Programme, Duke-NUS Medical School, 8 College Road, Singapore 169857; ^3^Singapore Immunology Network, A^∗^ STAR, 8A Biomedical Grove, Immunos, Singapore 138648; ^4^Clinical Imaging Research Centre, 14 Medical Drive, #B1-01, Yong Loo Lin School of Medicine, National University of Singapore, Singapore 117599

## Abstract

Hepatocellular carcinoma (HCC) is a notoriously difficult cancer to treat. The recent development of immune checkpoint inhibitors has revolutionised HCC therapy; however, successful response is only observed in a small percentage of patients. Biomarkers typically used to predict treatment response in other tumour types are ineffective in HCC, which arises in an immune-suppressive environment. However, imaging markers that measure changes in tumour infiltrating immune cells may supply information that can be used to determine which patients are responding to therapy posttreatment. We have evaluated [^18^F]AlF-mNOTA-GZP, a radiolabeled peptide targeting granzyme B, to stratify response to ICIs in a HEPA 1-tumours, a syngeneic model of HCC. Posttherapy, *in vivo* tumour retention of [^18^F]AlF-mNOTA-GZP was correlated to changes in tumour volume and tumour-infiltrating immune cells. [^18^F]AlF-mNOTA-GZP successfully stratified response to immune checkpoint inhibition in the syngeneic HEPA 1-6 model. FACS indicated significant changes in the immune environment including a decrease in immune suppressive CD4+ T regulatory cells and increases in tumour-associated GZB+ NK+ cells, which correlated well with tumour radiopharmaceutical uptake. While the immune response to ICI therapies differs in HCC compared to many other cancers, [^18^F]AlF-mNOTA-GZP retention is able to stratify response to ICI therapy associated with tumour infiltrating GZB+ NK+ cells in this complex tumour microenvironment.

## 1. Introduction

Hepatocellular carcinoma (HCC) is the most common primary liver cancer and is typically diagnosed at an advanced state [1]. First-line therapies for advanced HCC include systemic treatments such as the tyrosine kinase inhibitor sorafenib, but these are relatively ineffective and are associated with significant adverse effects [2]. The introduction of immune checkpoint inhibitor (ICI) therapies has been a major development in the treatment of advanced HCC, however, not all patients respond. The causes for this lack of response are poorly understood, biomarkers able to stratify response are an area of significant unmet clinical need, and accurate stratification of treatment response to ICIs is particularly important in HCC where many patients have existing chronic hepatic viral infections. Biomarkers associated with ICI efficacy in other cancer types appear to have little utility in HCC. Microsatellite instability (MSI) is a robust predictor of response to ICIs; however, the percentage of MSI-high cancers among HCC patients is very low [3, 4]. Likewise, tumour mutational burden (TMB) is a widely recognized biomarker of response but TMB high cancers are rare in HCC [5]. PD-L1 expression has also been demonstrated as a predictive biomarker of response in several cancer types; however, the efficacy of ICIs in HCC does not seem to be related to PD-L1 expression [6, 7]. In many cancers, the degree of tumour infiltrating lymphocytes (TILs) has been shown to be an accurate predictive of response to ICIs, and numerous biomarkers have been developed in an effort to quantify immune cell changes in the tumour microenvironment, usually targeting cytotoxic T cells [8–12]. However, the presence of TILs alone may not be enough for accurate stratification of response to ICI therapy in HCC where tumours have been found to harbour enriched populations of exhausted T cells, which show impaired immune surveillance, and a lack of response to ICIs [13]. Biomarkers of immune cell activation and tumouricidal activity may prove more successful [14]. Recently, noninvasive imaging of granzyme B, the serine protease released from active tumouricidal TILs, has been demonstrated to accurately stratify response to immunotherapy in syngeneic models of colon cancer [15–17]. In the current study, we have evaluated whether the granzyme B targeting peptide biomarker, [^18^F]AlF-mNOTA-GZP, is able to stratify responders to ICI therapy in the HEPA 1-6 syngeneic model of HCC, using flow cytometry to correlate tumour biomarker retention with tumour-associated immune cells.

## 2. Materials and Methods

### 2.1. [^18^F]AlF-mNOTA-GZP Radiochemistry

The synthesis of NOTA–*β*-Ala-Gly-Gly-Ile-Glu-Phe-Asp-CHO (mNOTA-GZP) and characterisation details have been described previously [16]. The radiosynthesis of [^18^F]AlF-mNOTA-GZP has also been described previously [16]. No-carrier-added aqueous [^18^F]fluoride ion was produced *via* the [^18^O(p,n)^18^F] nuclear reaction (GE PETtrace 860 cyclotron). Quality control analytical radio-HPLC was performed on a UFLC Shimazdu HPLC system equipped with dual-wavelength UV detector and a NaI/PMT-radiodetector (Flow-Ram, LabLogic). Radioactivity measurements were made with a CRC-55tPET dose calibrator (Capintec, USA). [^18^F]AlF-mNOTA-GZP was formulated as a colourless solution of 10% ethanol in saline (pH = 7.4) with a nondecay corrected radiochemical yield of 12-18%, a radiochemical purity of 99%, and molar activity of 50-81 GBq/*μ*mol (*n* = 3) after a total reaction and purification time of 50 minutes.

### 2.2. Animal Procedures

Animal procedures adhered to Institutional Animal Care and Use Committee Singapore regulations (IACUC No. 181399). C57/BL6 mice were acquired from In Vivos (Singapore) at 6-8 weeks of age, housed at standard room temperature with food and water provided ad libitum. HEPA 1-6 cells were purchased from ATCC, cultured in RPMI supplemented with 10% FBS, 100 U/mL penicillin, and 100 *μ*g/mL streptomycin at 37°C in a humidified atmosphere at 5% CO_2_. HEPA 1-6 cells were mixed 1 : 1 with Matrigel (Sigma) and injected subcutaneously into the right shoulder with a final concentration of 2X10 [6] per mouse. Tumour volumes were quantified using Vernier by callipers every 3 days from day 6 after tumour implantation. Tumour volumes were calculated as previously described using the modified ellipsoid formula (0.5 × Length × Width [2]).

Immune checkpoint inhibitor antibodies were purchased from Bio-X Cell (rat IgG2a isotype control (*α*-trinitrophenol mAb), rat IgG2a anti-mouse PD-1 (*α*PD1 mAb RMP1-14), and mouse IgG2b anti-mouse CTLA-4 (*α*CTLA4 mAb 9D9)) and dosed by intraperitoneal (i.p.) injection on days 6, 9, and 12 after tumour implantation (control IgG at 5 mg/kg, *α*PD1 monotherapy at 10 mg/kg, *α*CTLA4 monotherapy at 5 mg/kg or combined *α*PD1, and *α*CTLA4 therapy at 10 mg/kg and 5 mg/kg, respectively).

Response to therapy was determined using tumour growth inhibition (%TGI) on day 19 as previously described using the formula %TGI = (Vc − Vt)/(Vc − Vo) × 100 (Vc = control group tumour volume on day 19, Vt = treated group tumour volume on day 19, and Vo = treated group tumour volume on day 6 (Supplementary Table S3)).

### 2.3. PET-CT Imaging

Animals were imaged 12 days after tumour implantation as previously described using a Siemens Inveon PET-CT [15]. PET acquisitions were performed under isoflurane anaesthesia and acquired from 60–80 minutes postinjection (p.i.); CT was used for anatomical coregistration. Animal physiology was recorded using the Biovet physiological monitoring system. Calibrated images were reconstructed and analysed using FIJI and Amide software (version 10.3 Sourceforge). Volumes of interest (VOI) delineated by CT imaging were used to determine tissue uptake. Data are expressed as % of the injected dose per gram (%ID/g) of tumour tissue in the VOI.

### 2.4. Flow Cytometry

Tumours were removed post imaging and processed for flow cytometry as previously described [15]. Briefly, a single-cell suspension was generated from the tumour tissue and viable cells stained with the following antibodies for flow cytometry assessment on a BD FACSymphony; CD45 (clone 30-F11 BV570; Biolegend), CD3 (clone 500A2 BUV563; BD Biosciences), CD4 (clone RM4-5 BV650; BD Biosciences), CD8 (clone 53-6.7 BV510; BD Biosciences), CD25 (clone PC61 BUV395; BD Biosciences), F4/80 (clone BM8 biotin; Biolegend), CD206 (clone C068C2 PE-Cy7; Biolegend), Ly6C (clone HK1.4 BV605; Biolegend), NKp46 (clone 29A1.4 BUV737; BD Biosciences), CD11b (clone M1/70 APC-Cy7; Biolegend), I-A/I-E (clone M5/114.15.2 BV785; Biolegend), Ly6G (clone 1A8 BV480; BD Biosciences), FoxP3 (clone 150D AlexaFluor647; Biolegend), Fixable Live/Dead Blue (Invitrogen), Streptavidin BUV805 (BD Biosciences), PD-L1 (clone MIH5 BV421; BD Biosciences), CD107a (clone 1D4B FITC; Biolegend), CD170 (clone E50-2440 PECF594; BD Biosciences), Perforin (clone S16009A PE; Biolegend), CD11c (clone N418 BV711, Biolegend), and granzyme B (clone QA16A02 AlexaFluor700; Biolegend). Data was recompensated and analysed using FlowJo V10.5 software (FlowJo LLC).

### 2.5. Statistical Analysis

All data sets were assessed for normal distribution and analysed using GraphPad Prism 8.0.0. A Kruskal-Wallis 1-way ANOVA with a Dunn's posttest was used for analysis (*P* < 0.05 was considered statistically significant, and data are expressed as mean ± S.D.) unless otherwise indicated.

## 3. Results

### 3.1. [^18^F]AlF-NOTA-GZP Is Associated with Positive Therapy Response

**Figure 1:**
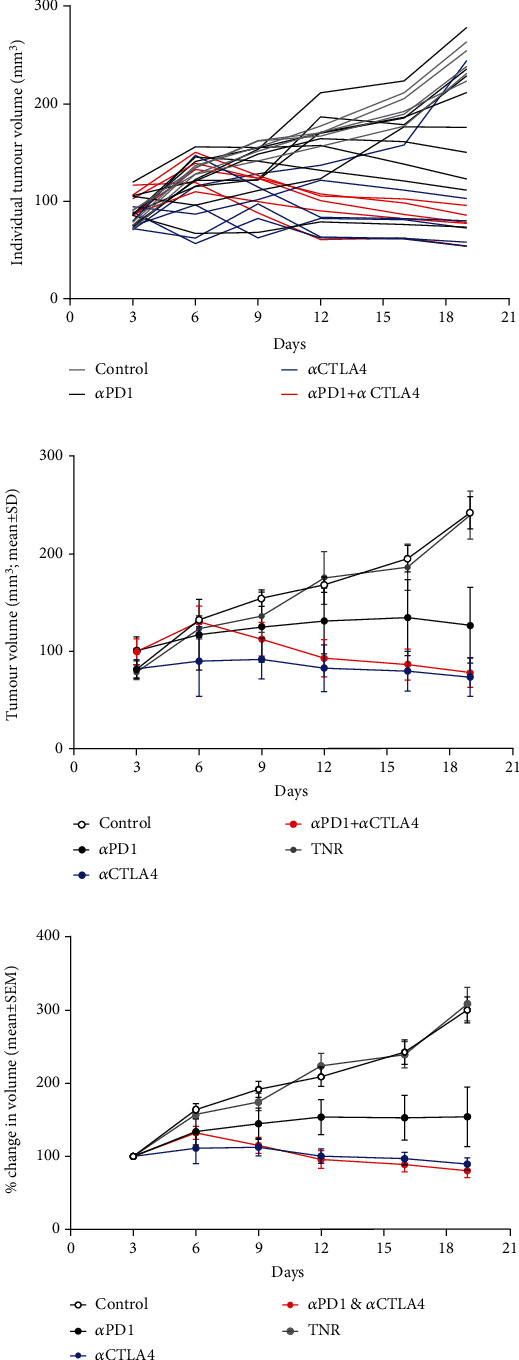
Graph displaying tumour volumes in response to administration of ICI therapy. Mice (*n* = 10 − 15) were i.p. treated with control IgG, *α*PD1 monotherapy, *α*CTLA4 monotherapy, or combined *α*PD1 + *α*CTLA4 on days 6, 9, and 12 posttumour implantation. (a) Individual HEPA 1-6 tumour volumes posttumour implantation. (b) Average tumour volume and (c) % change in tumour volume of HEPA 1-6 tumour-bearing mice on days 3, 6, 9, 12, 16, and 19 posttumour implantation. Data are shown postseparation of TNR group and represented as mean ± S.D. (TNR: treated nonresponder).

HEPA 1-6 liver tumour bearing mice were treated with control antibody or *α*PD1 monotherapy, *α*CTLA4 monotherapy or a combination of *α*PD1, and *α*CTLA4 and tumour volumes measured over the course of the study. Assessment of the tumour growth curves demonstrated normal distribution (Shapiro-Wilk p0.0588), and tumour volumes varied depending on the treatment regime and individual response (tumour volumes are depicted in Figures 1(a) and 1(b) and Supplementary Table S1, response rates in Supplementary Table S2). %TGI and tumour retention of [^18^F]AlF-NOTA-GZP are well correlated for all data (Pearson *r* = 0.706, ^∗∗∗^*P* < 0.001, *n* = 30, without post hoc manipulation). Successful response to therapy was determined based on tumour volumes at day 19 using a reductionist approach, which separated the tumours into treatment responders (TR comprising of both complete responders and partial responders) or treatment nonresponders (TNR) depending on final tumour volume at day 19. The cut-off value used to determine TRs was derived from the mean of the control group (>3 standard deviations from the mean control tumour volume on day 19, an approach that has been used before with less than 1% chance for a TR to be incorrectly assigned), those tumours with volumes less than 190 mm^3^ were designated as TR and all others as TNR (Supplementary Table S1). Overall % TGI and % response to therapy was greater in the combination-treated groups compared to the monotherapy treated groups.

**Figure 2:**
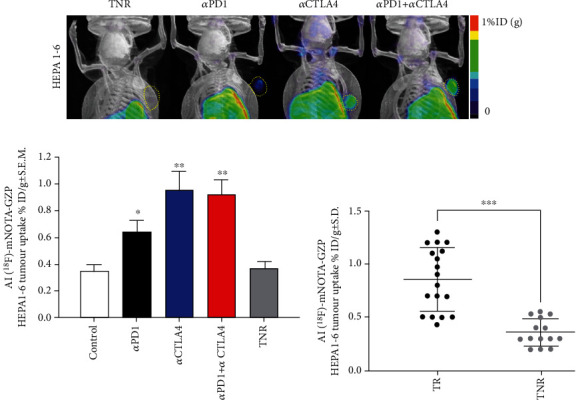
(a) Maximum intensity projection images depicting [^18^F]AlF-NOTA-GZP tumour retention. Yellow dotted areas indicate the HEPA 1-6 tumour border. Mice were injected with [^18^F]AlF-NOTA-GZP (~10 MBq intravenously) and static images acquired from 60-80 mins postinjection. (b) Graph showing differences in [^18^F]AlF-NOTA-GZP tumour retention from each treatment arm. [^18^F]AlF-NOTA-GZP tumour retention was significantly increased in *α*PD-1, *α*CTLA4 and combined αPD1 + *α*CTLA4 treatment arms when compared to treated nonresponders (TNR, *n* = 6 − 8 mice/group; ^∗^*P* < 0.05; ^∗∗^*P* < 0.01 comparing TR to TNR; data shown as mean%ID/g ± S.E.M.). (c) Individual values for [^18^F]AlF-NOTA-GZP tumour retention in TRs and TNRs (^∗∗∗^*P* < 0.001, data shown as individual %ID/g).

Tumour retention of [^18^F]AlF-NOTA-GZP was heterogeneous across the different treatment arms (Figure 2(a)), and the control group and TNRs displayed low tumour retention whereas tumour retention in the TR groups was significantly higher; *α*PD1 (^∗^*P* < 0.05, *n* = 5), *α*CTLA4 (^∗∗^*P* < 0.01, *n* = 7), and combined *α*PD1 + *α*CTLA4 (^∗∗^*P* < 0.01, *n* = 8), when compared to the TNR group (*n* = 7, Table 1 and Figure 2).

### 3.2. [^18^F]AlF-NOTA-GZP Tumour Retention Is Dependent on Granzyme B Expression on Tumour-Infiltrating NK Cells

**Figure 3:**
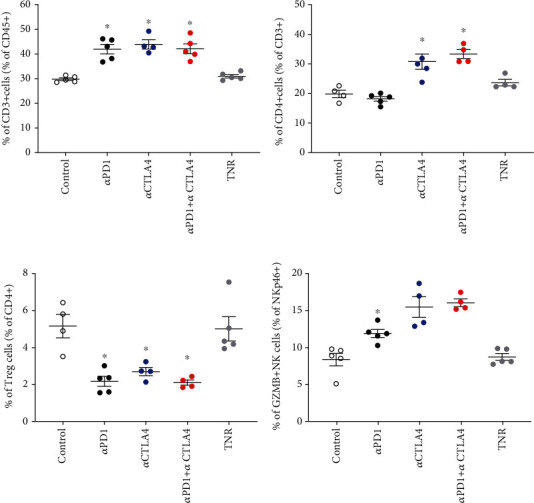
Multicolour flow cytometry analysis of HEPA 1-6 tumour-associated immune cells after treatment. Percentages of (a) CD3+ T cells relative to CD45+ cells, (b) CD4+ TILS relative to total CD3+ T cells, (c) CD4+ Treg cells relative to total CD4+ cells, and (d) GZB+ NK+ cells relative to total NK+ cells. Data are shown as individual values with mean ± S.D. and are representative of *n* = 4 − 5 mice/group. ^∗^*P* < 0.05; ^∗∗^*P* < 0.01 compared to TNR.

We analysed the changes in HEPA 1-6 tumour uptake of different infiltrating immune cells in response to therapy, and t-SNE and Rphenoptype clustering were used to determine immunophenotypic changes across the different treatment arms comparing each TR group against the TNR group (Figure 3). Many immunophenotypic changes were detected between the responsive tumours and TNRs (Supplementary Table S4). Significant changes were attributed to CD3+ and CD4+ T cells and GZB+ NK+ cells (Figure 3 and Table 2). Overall, the strongest correlation was observed between [^18^F]AlF-NOTA-GZP and GZB+ NK+ cells (Supplementary Figure 1, Pearson *r* = 0.859, ^∗∗∗^*P* < 0.001, *n* = 23).

## 4. Discussion

The liver is a tolerogenic organ, able to regulate immune responses to a constant stream of antigens from the gut [18], this immune-suppressive environment, however, is detrimental for tumorigenesis. Chronic inflammation caused by viral infections or long-term damage (alcoholic cirrhosis, steatohepatitis, or nonalcoholic fatty liver disease) drives the development of HCC [19]. Chronic inflammation also leads to key players in the immune system becoming suppressed, resulting in incomplete activation of T cells and immune exhaustion [20, 21]. Despite such a chronically immunosuppressed environment, ICI therapy has shown great promise in HCC with recent clinical trials showing the benefit of anti-PD1 or anti-CTLA4 treatment alone or in combination with antiangiogenic therapy (CHECKMATE 40, KEYNOTE 240, and KEYNOTE 224) [1, 3, 19, 22–26]. Overall survival remains the primary endpoint in HCC clinical trials; however, imaging-based assessment allows for estimations of objective response rates (ORR) used to identify strong early signals of efficacy [2]. However, assessment of treatment response in HCC is complex, the lack of tumour shrinkage with some effective therapies and the coexistence of cancer and cirrhosis in HCC patients makes conventional RECIST-based imaging assessment unreliable [27, 28]. Imaging biomarkers that provide information about changes in the tumoural microenvironment after treatment may provide reliable markers of objective response. Previous studies have shown that GZB-targeting peptides can stratify ICI therapeutic response in syngeneic models of colon cancer [16, 17]; however, this has not been demonstrated in a syngeneic HCC before. HCC tumour retention of [^18^F]AlF-mNOTA-GZP was significantly higher in treatment responsive tumours compared to TNRs (Table 1, Figure 2(b), Supplementary Figure 2); however, the immune mechanisms involved were very different to other syngeneic cancers. In previous studies, syngeneic colon cancers responding to anti-PD1, CTLA4, or combination therapy displayed increases in CD8+ and GZB+ CD8+ cells and decreases in F4/80 myeloid cells [15, 16]. Treatment responsive HCCs, however, responded via different immune pathways, with no significant changes observed in CD8+ or myeloid cell populations but instead a significant increase in tumour infiltrating GZB+ NK+ cells and a concomitant decrease in CD4+ T-regulatory cells. NK cells make up a significant part of the intrahepatic lymphocyte population and are positively correlated with HCC patients' survival and prognosis [29, 30]. NK cells are involved in the innate and adaptive immune response through cross talk with dendritic cells and T cells and are rapidly gaining popularity as a target for the development of new ICIs [31, 32]. The presence of the CD4+ T-reg cells (CD4+/FOXP3+) has been linked with poor prognosis in HCC and a reduced response to ICI therapy [33, 34] so decreases in tumour infiltration of CD4+ Treg cells may ease the immunosuppressive environment allowing immune recognition.

Recently, new granzyme B targeting tracers have been developed whose tumour retention is dependent on granzyme B proteolytic activity as opposed to the current aldehyde tracer which quantifies the amount of biochemically active GZMB molecules at the tumour [35]. One of the documented shortcomings of the aldehyde granzyme B tracers is low tumour retention, which may be masked in tumours originating in tissues with higher nonspecific retention including liver and kidneys. The new granzyme B tracer, dependent on proteolytic activity, shows higher tumour retention, but due to its considerable size is cleared much more slowly from blood, leading to higher background. It will be interesting to see how these different radiopharmaceuticals can be used to provide complementary information to stratify therapy response in future.

Overall, [^18^F]AlF-mNOTA-GZP demonstrates the ability to accurately stratify ICI response in HCCs, showing that granzyme B radiopharmaceuticals can be used across multiple tumour types, including those arising in immune suppressive environments. While the immune response to ICI therapies differs in HCC compared to many other cancers, granzyme B may still be a potential biomarker for immunotherapy efficacy in liver cancer providing useful information to help improve patient management.

## Figures and Tables

**Table 1:** Table showing HEPA 1-6 tumour retention of [^18^F]AlF-NOTA-GZP after completion of ICI treatment. Data are shown as mean%ID/g ± S.D. and are separated into control, treatment responders (TR), and treatment nonresponders (TNR) (*n* = 10 mice/group; ^∗^*P* < 0.05; ^∗∗^*P* < 0.01, comparing TR to TNR).

	[^18^F]AlF-NOTA-GZP retention in HEPA 1-6 tumours
Control	0.35 ± 0.13
Treatment responders (TR) *α*PD1	0.64 ± 0.19^∗^
*α*CTLA4	0.95 ± 0.31^∗∗^
*α*PD1 + *α*CTLA4	0.93 ± 0.31^∗∗^
Treatment nonresponders (TNR)	0.37 ± 0.13

**Table 2:** Table showing HEPA 1-6 tumour infiltrating immune cells after completion of ICI treatment. Data are shown as mean%of cells ± S.D. and are separated into control, treatment responders (TR), and treatment nonresponders (TNR) and are representative of *n* = 5 − 10 mice/group, ^∗^*P* < 0.05; ^∗∗^*P* < 0.01, comparing TR to TNR.

	Immune cell subsets associated with HEPA 1-6 tumours			
	CD3+ % of CD45+	CD4+ % of CD3+	CD4+ Treg % of CD4+	GZB+ NK+ % of NK+
Control	30.10 ± 1.17	19.85 ± 2.50	5.17 ± 1.27	8.39 ± 1.90
TR *α*PD1	40.80 ± 6.43^∗^	18.18 ± 1.81	2.18 ± 0.61^∗^	11.92 ± 1.22^∗^
*α*CTLA4	43.95 ± 3.76^∗^	31.78 ± 4.62^∗^	2.71 ± 0.45^∗^	15.50 ± 2.81^∗^
*α*PD1 + *α*CTLA4	41.56 ± 5.42^∗^	33.35 ± 3.01^∗^	2.11 ± 0.28^∗^	16.08 ± 1.04^∗∗^
TNR	30.98 ± 1.54	23.68 ± 2.16	5.02 ± 1.47	8.74 ± 1.02

## Data Availability

All data is available on request to the corresponding author.

## Supplementary Materials

Supplementary 1 Supplementary Table S1: table showing the tumour volumes at each date across the different treatment arms postinduction of checkpoint inhibitor monotherapy or combination therapy. Data are shown as mm^3^mean ± S.D. and are representative of *n* = 5 − 10 mice/group.

Supplementary 2 Supplementary Table S2: summary of ICI treatment responders (TR) and treatment nonresponders (TNR) across all therapy arms.

Supplementary 3 Supplementary Table S3: summary of %TGI across all therapy arms.

Supplementary 4 Supplementary Table S4: table showing the tumour associated immune cell populations from HEPA 1-6 tumour-bearing mice at day 14 postinduction of checkpoint inhibitor monotherapy or combination therapy. (a) Percentages of CD3+, CD4+, CD4+ Teff, CD4+ Treg, CD8+, and GZB+ CD8+ immune cell subpopulations. (b) Percentages of NK+, GZB+ NK+, Eos, GZB+ Eos, Ly6G+, and Ly6C+ immune cell subpopulations. (c) Percentages of F4/80+, F4/80+ CD206+, and F4/80+ CD206-immune cell subpopulations are shown across control groups, treatment responders (TR), and treatment nonresponders (TNR) across all treatment arms. Data are shown as mean%of cells ± S.D. and are representative of *n* = 4 − 6 mice/group, ^∗^*P* < 0.05; ^∗∗^*P* < 0.01, ^∗∗∗^*P* < 0.001, comparing TR to TNR.

Supplementary 5 Supplementary Figure S1: a linear regression model of [^18^F]AlF-mNOTA-GZP tumour uptake versus individual GZB+ NK+ TILs with Pearson's correlation and corresponding *r*-value.

Supplementary 6 Supplementary Figure S2: ex vivo biodistribution analysis of [^18^F]AlF-mNOTA-GZP retention in selected organs. ICI treated responder (TR, black) and treated nonresponder (TNR, white) animals were sacrificed 80 min postinjection, tissues excised, weighed, and radioactivity quantified using a Wallac gamma counter. Bars represent the mean of 5 animals ± SEM, ^∗^*P* < 0.05.

## References

[1] Wang H., Li W. (2021). Recent update on comprehensive therapy for advanced hepatocellular carcinoma. *World Journal of Gastrointestinal Oncology*.

[2] Llovet J. M., Villanueva A., Lachenmayer A., Finn R. S. (2015). Advances in targeted therapies for hepatocellular carcinoma in the genomic era. *Nature Reviews. Clinical Oncology*.

[3] D'Alessio A., Rimassa L., Cortellini A., Pinato D. J. (2021). PD-1 blockade for hepatocellular carcinoma: current research and future prospects. *Journal of Hepatocellular Carcinoma*.

[4] Eso Y., Shimizu T., Takeda H., Takai A., Marusawa H. (2020). Microsatellite instability and immune checkpoint inhibitors: toward precision medicine against gastrointestinal and hepatobiliary cancers. *Journal of Gastroenterology*.

[5] Marabelle A., Fakih M., Lopez J. (2020). Association of tumour mutational burden with outcomes in patients with advanced solid tumours treated with pembrolizumab: prospective biomarker analysis of the multicohort, open-label, phase 2 KEYNOTE-158 study. *The Lancet Oncology*.

[6] el-Khoueiry A. B., Sangro B., Yau T. (2017). Nivolumab in patients with advanced hepatocellular carcinoma (CheckMate 040): an open-label, non-comparative, phase 1/2 dose escalation and expansion trial. *The Lancet*.

[7] Inarrairaegui M., Melero I., Sangro B. (2018). Immunotherapy of hepatocellular carcinoma: facts and hopes. *Clinical Cancer Research*.

[8] Larimer B. M., Wehrenberg-Klee E., Caraballo A., Mahmood U. (2016). Quantitative CD3 PET imaging predicts tumor growth response to anti-CTLA-4 therapy. *Journal of Nuclear Medicine*.

[9] Seo J. W., Tavaré R., Mahakian L. M. (2018). CD8+T-Cell density imaging with64Cu-Labeled cys-diabody informs immunotherapy protocols. *Clinical Cancer Research*.

[10] Tavaré R., Escuin-Ordinas H., Mok S. (2016). An effective immuno-PET imaging method to monitor CD8-dependent responses to immunotherapy. *Cancer Research*.

[11] Tavare R., McCracken M. N., Zettlitz K. A. (2014). Engineered antibody fragments for immuno-PET imaging of endogenous CD8+ T cells in vivo. *Proceedings of the National Academy of Sciences of the United States of America*.

[12] Tavaré R., McCracken M. N., Zettlitz K. A. (2015). Immuno-PET of murine T cell reconstitution postadoptive stem cell transplantation using anti-CD4 and anti-CD8 cys-diabodies. *Journal of Nuclear Medicine*.

[13] Hsu C. L., Ou D. L., Bai L. Y. (2021). Exploring markers of exhausted CD8 T cells to predict response to immune checkpoint inhibitor therapy for hepatocellular carcinoma. *Liver Cancer*.

[14] Goggi J. L., Hartimath S. V., Hwang Y. (2020). Examining immunotherapy response using multiple radiotracers. *Molecular Imaging and Biology*.

[15] Goggi J. L., Hartimath S. V., Xuan T. Y. (2021). Granzyme B PET imaging of combined chemotherapy and immune checkpoint inhibitor therapy in colon cancer. *Molecular Imaging and Biology*.

[16] Goggi J. L., Tan Y. X., Hartimath S. V. (2020). Granzyme B PET imaging of immune checkpoint inhibitor combinations in colon cancer phenotypes. *Molecular Imaging and Biology*.

[17] Larimer B. M., Wehrenberg-Klee E., Dubois F. (2017). Granzyme B PET imaging as a predictive biomarker of immunotherapy response. *Cancer Research*.

[18] Trivedi P. J., Adams D. H. (2016). Gut-liver immunity. *Journal of Hepatology*.

[19] Fu Y., Liu S., Zeng S., Shen H. (2019). From bench to bed: the tumor immune microenvironment and current immunotherapeutic strategies for hepatocellular carcinoma. *Journal of Experimental & Clinical Cancer Research*.

[20] Horst A. K., Neumann K., Diehl L., Tiegs G. (2016). Modulation of liver tolerance by conventional and nonconventional antigen-presenting cells and regulatory immune cells. *Cellular & Molecular Immunology*.

[21] Kanterman J., Sade-Feldman M., Baniyash M. (2012). New insights into chronic inflammation-induced immunosuppression. *Seminars in Cancer Biology*.

[22] Fulgenzi C. A. M., Talbot T., Murray S. M. (2021). Immunotherapy in hepatocellular carcinoma. *Current Treatment Options in Oncology*.

[23] Pinato D. J., Cortellini A., Sukumaran A. (2021). PRIME-HCC: phase Ib study of neoadjuvant ipilimumab and nivolumab prior to liver resection for hepatocellular carcinoma. *BMC Cancer*.

[24] Prieto J., Melero I., Sangro B. (2015). Immunological landscape and immunotherapy of hepatocellular carcinoma. *Nature Reviews. Gastroenterology & Hepatology*.

[25] Tagliamonte M., Petrizzo A., Tornesello M. L., Ciliberto G., Buonaguro F. M., Buonaguro L. (2016). Combinatorial immunotherapy strategies for hepatocellular carcinoma. *Current Opinion in Immunology*.

[26] Vogel A., Rimassa L., Sun H. C. (2021). Comparative efficacy of atezolizumab plus bevacizumab and other treatment options for patients with unresectable hepatocellular carcinoma: a network meta-analysis. *Liver Cancer*.

[27] Forner A., Ayuso C., Varela M. (2009). Evaluation of tumor response after locoregional therapies in hepatocellular carcinoma. *Cancer*.

[28] Lencioni R., Llovet J. M. (2010). Modified RECIST (mRECIST) assessment for hepatocellular carcinoma. *Seminars in Liver Disease*.

[29] Kalathil S. G., Thanavala Y. (2021). Natural killer cells and T cells in hepatocellular carcinoma and viral hepatitis: current status and perspectives for future immunotherapeutic approaches. *Cell*.

[30] Mantovani S., Oliviero B., Varchetta S., Mele D., Mondelli M. U. (2020). Natural killer cell responses in hepatocellular carcinoma: implications for novel immunotherapeutic approaches. *Cancers*.

[31] Bozward A. G., Warricker F., Oo Y. H., Khakoo S. I. (2021). Natural killer cells and regulatory T cells cross talk in hepatocellular carcinoma: exploring therapeutic options for the next decade. *Frontiers in Immunology*.

[32] Khan M., Arooj S., Wang H. (2020). NK cell-based immune checkpoint inhibition. *Frontiers in Immunology*.

[33] Tian M. X., Liu W. R., Wang H. (2019). Tissue-infiltrating lymphocytes signature predicts survival in patients with early/intermediate stage hepatocellular carcinoma. *BMC Medicine*.

[34] Shi C., Chen Y., Chen Y., Yang Y., Bing W., Qi J. (2019). CD4^+^ CD25^+^ regulatory T cells promote hepatocellular carcinoma invasion via TGF-*β*1-induced epithelial–mesenchymal transition. *Oncotargets and Therapy*.

[35] Zhao N., Bardine C., Lourenço A. L. (2021). In vivo measurement of granzyme proteolysis from activated immune cells with PET. *ACS Central Science*.

