# Supplementary material for: Granzyme B PET Imaging Stratifies Immune Checkpoint Inhibitor Response in Hepatocellular Carcinoma
Source: Mol Imaging. 2021 Dec 9;2021:9305277. doi: 10.1155/2021/9305277 (PMC9328186; doi:10.1155/2021/9305277)
Supplement: Supplementary 1 — Supplementary Table S1: table showing the tumour volumes at each date across the different treatment arms postinduction of checkpoint inhibitor monotherapy or combination therapy. Data are shown as mm3mean ± S.D. and are representative of n = 5 − 10 mice/group. [file 9305277.f1.docx]

| **Day** | **Control** | **αPD1** | **αCTLA4** | **αPD1 & αCTLA4** | **TNR** |
| --- | --- | --- | --- | --- | --- |
| **3** | 81.52 ± 8.93 | 100.64 ± 14.42 | 82.07 ± 9.57 | 99.62 ± 13.35 | 78.60 ± 7.64 |
| **6** | 132.42 ± 4.08 | 117.16 ± 36.32 | 89.96 ± 36.09 | 130.35 ± 15.98 | 123.21 ± 10.49 |
| **9** | 154.61 ± 8.59 | 124.89 ± 35.91 | 91.81 ± 20.04 | 112.45 ± 17.40 | 136.20 ± 16.63 |
| **14** | 168.41 ± 7.62 | 131.19 ± 33.81 | 82.76 ± 24.01 | 93.00 ± 19.20 | 175.28 ± 27.17 |
| **16** | 195.26 ± 13.61 | 134.70 ± 38.98 | 79.74 ± 20.47 | 86.44 ± 15.84 | 186.48 ± 23.77 |
| **19** | 242.45 ± 16.63 | 126.87 ± 38.89 | 73.53 ± 19.59 | 78.28 ± 15.34 | 239.94 ± 24.71 |

**Supplementary Table S1.** Table showing the tumour volumes at each date across the different treatment arms post-induction of checkpoint inhibitor monotherapy or combination therapy. Data are shown as mm^3^ mean ± S.D. and are representative of n=5-10 mice/ group.
