# Supplementary material for: Granzyme B PET Imaging Stratifies Immune Checkpoint Inhibitor Response in Hepatocellular Carcinoma
Source: Mol Imaging. 2021 Dec 9;2021:9305277. doi: 10.1155/2021/9305277 (PMC9328186; doi:10.1155/2021/9305277)
Supplement: Supplementary 2 — Supplementary Table S2: summary of ICI treatment responders (TR) and treatment nonresponders (TNR) across all therapy arms. [file 9305277.f2.docx]

|  | **Treatment Responders (TR)/**  **Treatment Non-Responders (TNR)**  **(No. mice)** |
| --- | --- |
| **ICI Treatment** | **HEPA 1-6** |
| Control | 0/5 |
| αPD1 | 5/10 |
| αCTLA4 | 7/10 |
| αPD1 + αCTLA4 | 8/10 |

Supplementary Table S2. Summary of ICI treatment responders (TR) and treatment non-responders (TNR) across all therapy arms.
