# Supplementary material for: Granzyme B PET Imaging Stratifies Immune Checkpoint Inhibitor Response in Hepatocellular Carcinoma
Source: Mol Imaging. 2021 Dec 9;2021:9305277. doi: 10.1155/2021/9305277 (PMC9328186; doi:10.1155/2021/9305277)
Supplement: Supplementary 3 — Supplementary Table S3: summary of %TGI across all therapy arms. [file 9305277.f3.docx]

|  | **% Tumour Growth Inhibition** |
| --- | --- |
| **ICI Treatment** | **HEPA 1-6** |
| Control | NA |
| αPD1 | 81.1 ± 23.6 |
| αCTLA4 | 105.3 ± 11.1 |
| αPD1 + αCTLA4 | 116.2 ± 19.7 |
| TNR | 1.7 ± 14.8 |

Supplementary Table S3. Summary of %TGI across all therapy arms.
