# Supplementary material for: Granzyme B PET Imaging Stratifies Immune Checkpoint Inhibitor Response in Hepatocellular Carcinoma
Source: Mol Imaging. 2021 Dec 9;2021:9305277. doi: 10.1155/2021/9305277 (PMC9328186; doi:10.1155/2021/9305277)
Supplement: Supplementary 4 — Supplementary Table S4: table showing the tumour associated immune cell populations from HEPA 1-6 tumour-bearing mice at day 14 postinduction of checkpoint inhibitor monotherapy or combination therapy. (a) Percentages of CD3+, CD4+, CD4+ Teff, CD4+ Treg, CD8+, and GZB+ CD8+ immune cell subpopulations. (b) Percentages of NK+, GZB+ NK+, Eos, GZB+ Eos, Ly6G+, and Ly6C+ immune cell subpopulations. (c) Percentages of F4/80+, F4/80+ CD206+, and F4/80+ CD206-immune cell subpopulations are shown across control groups, treatment responders (TR), and treatment nonresponders (TNR) across all treatment arms. Data are shown as mean%of cells ± S.D. and are representative of n = 4 − 6 mice/group, ∗P < 0.05; ∗∗P < 0.01, ∗∗∗P < 0.001, comparing TR to TNR. [file 9305277.f4.docx]

**A**

|  | **CD3+ %**  **of CD45+** | **CD4+ %**  **of CD3+** | **CD4+ Teff %**  **of CD4+** | **CD4+ Treg %**  **of CD4+** | **CD8+ %**  **of CD3+** | **GZB+ CD8+ %**  **of CD8+** |
| --- | --- | --- | --- | --- | --- | --- |
| **Control** | 30.10 ± 1.17 | 19.85 ± 2.50 | 91.50 ± 2.18 | 5.17 ± 1.27 | 72.73 ± 2.88 | 30.18 ± 4.07 |
| **TR**  **αPD1** | 40.80 ± 6.43* | 18.18 ± 1.81 | 93.86 ± 1.60 | 2.18 ± 0.61* | 77.64 ± 2.19 | 21.54 ± 1.80 |
| **αCTLA4** | 43.95 ± 3.76* | 31.78 ± 4.62* | 96.13 ± 0.70 | 2.71 ± 0.45* | 66.88 ± 6.19 | 20.22 ± 5.54 |
| **αPD1 + αCTLA4** | 41.56 ± 5.42* | 33.35 ± 3.01* | 95.45 ± 0.54 | 2.11 ± 0.28* | 63.20 ± 3.00 | 18.23 ± 3.19 |
| **TNR** | 30.98 ± 1.54 | 23.68 ± 2.16 | 94.64 ± 1.27 | 5.02 ± 1.47 | 68.42 ± 4.57 | 24.18 ± 2.34 |

**B**

|  | **NK+ %**  **of CD45+** | **GZB+ NK+ %**  **of NK+** | **Eos %**  **of CD45+** | **GZB+ Eos %**  **of Eos+** | **Ly6G+ %**  **of CD45+** | **Ly6C+ %**  **of CD45+** |
| --- | --- | --- | --- | --- | --- | --- |
| **Control** | 1.14 ± 0.28 | 8.39 ± 1.90 | 50.53 ± 8.76 | 85.60 ± 5.49 | 1.56 ± 2.04 | 2.70 ± 1.18 |
| **TR**  **αPD1** | 1.08 ± 0.37 | 11.92 ± 1.22* | 42.24 ± 7.11 | 78.60 ± 2.19 | 3.12 ± 1.99 | 3.94 ± 0.55 |
| **αCTLA4** | 1.25 ± 0.53 | 15.50 ± 2.81* | 39.23 ± 6.03 | 75.43 ± 9.11 | 2.83 ± 1.65 | 2.23 ± 0.61 |
| **αPD1 + αCTLA4** | 1.22 ± 0.30 | 16.08 ± 1.04** | 47.65 ± 4.99 | 80.13 ± 10.16 | 1.56 ± 1.60 | 1.91 ± 1.12 |
| **TNR** | 1.23 ± 0.42 | 8.74 ± 1.02 | 48.16 ± 6.26 | 83.46 ± 7.50 | 1.84 ± 1.21 | 3.16 ± 1.62 |

**C**

|  | **F4/80+ %**  **of CD45+** | **F4/80+ CD206+%**  **of F4/80+** | **F4/80+ CD206- %**  **of F4/80+** |
| --- | --- | --- | --- |
| **Control** | 3.55 ± 1.17 | 95.90 ± 3.01 | 1.51 ± 0.80 |
| **TR**  **αPD1** | 2.82 ± 0.67 | 94.28 ± 3.83 | 2.39 ± 1.28 |
| **αCTLA4** | 5.02 ± 1.41 | 95.50 ± 2.82 | 2.16 ± 1.32 |
| **αPD1 + αCTLA4** | 6.95 ± 3.56 | 97.80 ± 1.13 | 2.00 ± 0.62 |
| **TNR** | 3.71 ± 0.36 | 96.42 ± 2.55 | 2.05 ± 1.46 |

**Supplementary Table S4.** Table showing the tumour associated immune cell populations from HEPA 1-6 tumour-bearing mice at day 14 post-induction of checkpoint inhibitor monotherapy or combination therapy. **A**. Percentages of CD3+, CD4+, CD4+ Teff, CD4+ Treg, CD8+ and GZB+ CD8+ immune cell subpopulations **B**. Percentages of NK+, GZB+ NK+, Eos, GZB+ Eos, Ly6G+ and Ly6C+ immune cell subpopulations, **C**. Percentages of F4/80+, F4/80+ CD206+ and F4/80+ CD206- immune cell subpopulations are shown across control groups, treatment responders (TR) and treatment non-responders (TNR) across all treatment arms. Data are shown as mean % of cells ± S.D. and are representative of n=4-6 mice/ group, * *P*<0.05; ** *P*<0.01, *** *P*<0.001, comparing TR to TNR.
