# Supplementary figures and images for: Granzyme B PET Imaging Stratifies Immune Checkpoint Inhibitor Response in Hepatocellular Carcinoma
Source: Mol Imaging. 2021 Dec 9;2021:9305277. doi: 10.1155/2021/9305277 (PMC9328186; doi:10.1155/2021/9305277)

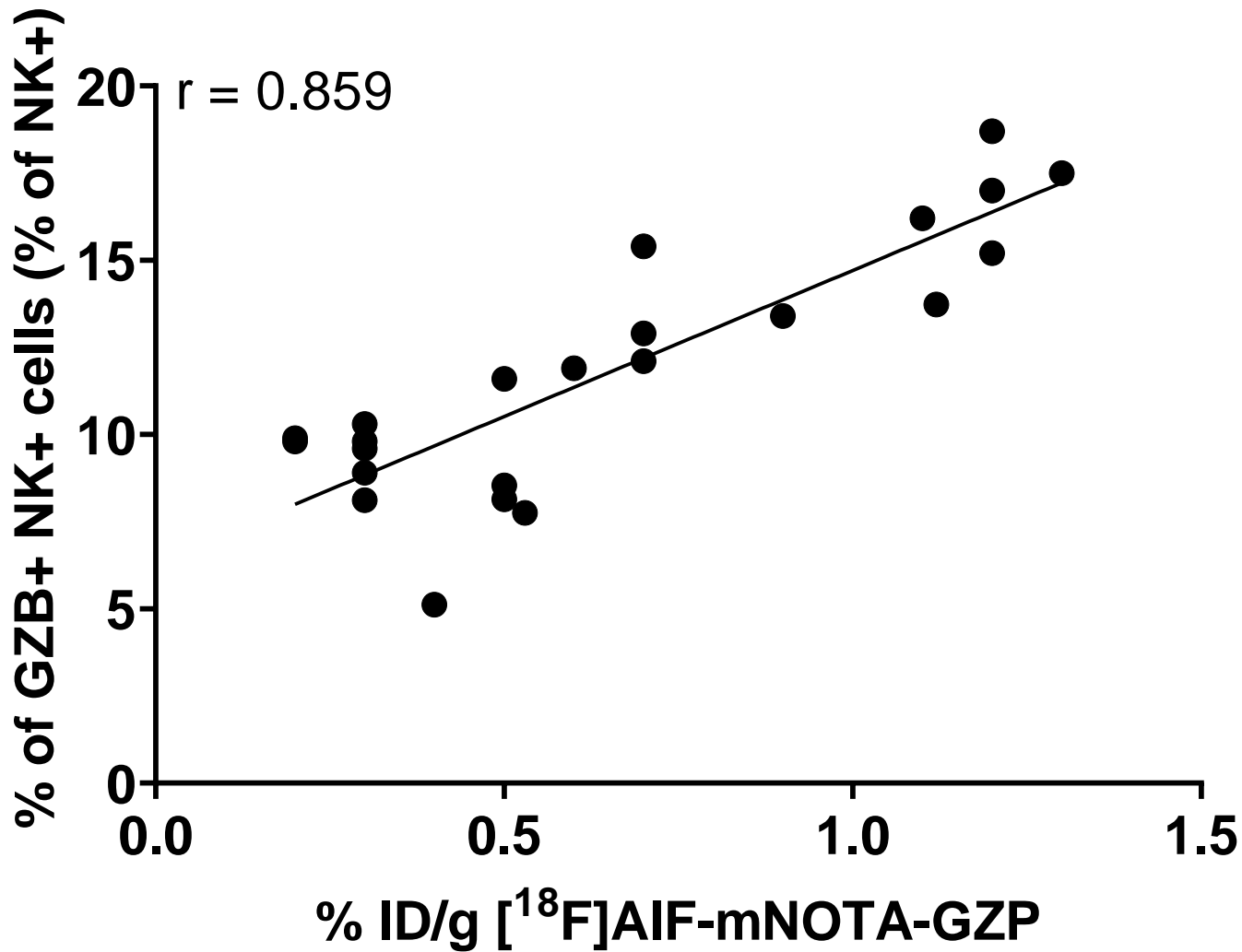

Supplement: Supplementary 5 — Supplementary Figure S1: a linear regression model of [18F]AlF-mNOTA-GZP tumour uptake versus individual GZB+ NK+ TILs with Pearson's correlation and corresponding r-value. [file 9305277.f5.pdf]

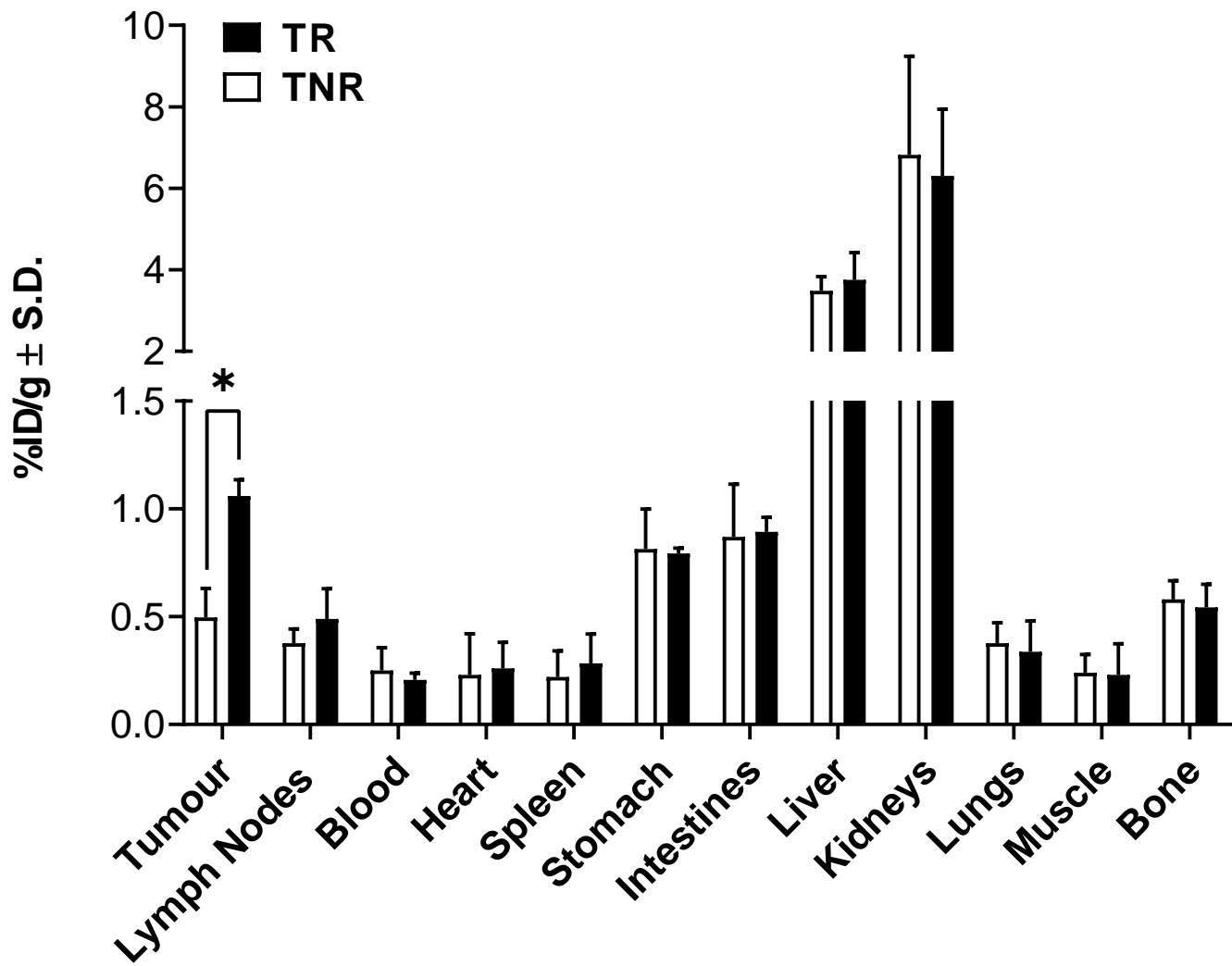

Supplement: Supplementary 6 — Supplementary Figure S2: ex vivo biodistribution analysis of [18F]AlF-mNOTA-GZP retention in selected organs. ICI treated responder (TR, black) and treated nonresponder (TNR, white) animals were sacrificed 80 min postinjection, tissues excised, weighed, and radioactivity quantified using a Wallac gamma counter. Bars represent the mean of 5 animals ± SEM, ∗P < 0.05. [file 9305277.f6.pdf]
